# Identification of genes associated with nitrogen-use efficiency by genome-wide transcriptional analysis of two soybean genotypes

**DOI:** 10.1186/1471-2164-12-525

**Published:** 2011-10-26

**Authors:** Qing N Hao, Xin A Zhou, Ai H Sha, Cheng Wang, Rong Zhou, Shui L Chen

**Affiliations:** 1 Institute of Oil Crops Research, Chinese Academy of Agriculture Sciences, Wuhan 430062, China; 2 Chinese Academy of Agricultural Sciences/Key Laboratory for Biological Sciences of Oil Crops, Ministry of Agriculture, Wuhan 430062, China; 3 Graduate School of Chinese Academy of Agriculture Sciences, Beijing 100081, China; 4 Graduate School of Nanjing Agricultural University, Nanjing, 210095, China

## Abstract

**Background:**

Soybean is a valuable crop that provides protein and oil. Soybean requires a large amount of nitrogen (N) to accumulate high levels of N in the seed. The yield and protein content of soybean seeds are directly affected by the N-use efficiency (NUE) of the plant, and improvements in NUE will improve yields and quality of soybean products. Genetic engineering is one of the approaches to improve NUE, but at present, it is hampered by the lack of information on genes associated with NUE. Solexa sequencing is a new method for estimating gene expression in the transcription level. Here, the expression profiles were analyzed between two soybean varieties in N-limited conditions to identify genes related to NUE.

**Results:**

Two soybean genotypes were grown under N-limited conditions; a low-N-tolerant variety (No.116) and a low-N-sensitive variety (No.84-70). The shoots and roots of soybeans were used for sequencing. Eight libraries were generated for analysis: 2 genotypes × 2 tissues (roots and shoots) × 2 time periods [short-term (0.5 to 12 h) and long-term (3 to 12 d) responses] and compared the transcriptomes by high-throughput tag-sequencing analysis. 5,739,999, 5,846,807, 5,731,901, 5,970,775, 5,476,878, 5,900,343, 5,930,716, and 5,862,642 clean tags were obtained for the eight libraries: L1, 116-shoot short-term; L2 84-70-shoot short-term; L3 116-shoot long-term; L4 84-70-shoot long-term; L5 116-root short-term; L6 84-70-root short-term; L7 116-root long-term;L8 84-70-root long-term; these corresponded to 224,154, 162,415, 191,994, 181,792, 204,639, 206,998, 233,839 and 257,077 distinct tags, respectively. The clean tags were mapped to the reference sequences for annotation of expressed genes. Many genes showed substantial differences in expression among the libraries. In total, 3,231genes involved in twenty-two metabolic and signal transduction pathways were up- or down-regulated. Twenty-four genes were randomly selected and confirmed their expression patterns by quantitative RT-PCR; Twenty-one of the twenty-four genes showed expression patterns consistent with the Digital Gene Expression (DGE) data.

**Conclusions:**

A number of soybean genes were differentially expressed between the low-N-tolerant and low-N-sensitive varieties under N-limited conditions. Some of these genes may be candidates for improving NUE. These findings will help to provide a detailed understanding of NUE mechanisms, and also provide a basis for breeding soybean varieties that are tolerant to low-N conditions.

## Background

Plants require large amounts of nitrogen (N) for their growth and survival [1]. This N accounts for approximately 2% of total plant dry matter. N is a necessary component of proteins, enzymes, and metabolic products involved in the synthesis and transfer of energy. At present, the increase in investment in agriculture is mainly due to the use of nitrogen fertilizer because it directly affects yield. Nitrogen fertilizer consumption has been increasing since the early 1960's, and has stabilized slightly over the last decade [2]. Plants can only use approximately 30-40% of the applied N, and more than 40% of the N fertilizer is lost via leakage into the atmosphere, groundwater, lakes and rivers. Such leakage results in serious environmental pollution [3]. The United Nations Environment Programme recently reported that, N pollution, water shortages and global warming are the main global threats [4]. Improving crop and N management is required to optimize crop production and reduce environmental risks due to N losses.

Improving N-use efficiency (NUE) by genetic improvement is necessary for the development of agriculture. NUE comprises assimilation efficiency, which involves N uptake and assimilation, and utilization efficiency, which involves N remobilization. The mechanisms regulating these processes are complex, but it is vital that they are well understood to improve NUE in plants [5]. To study the whole physiological process, the plants grown under low- and high-nitrogen conditions were compared, and the genes, proteins, and other metabolites that played roles in the various steps of nitrogen uptake, assimilation, and remobilization were described in detail [6]. There were significant differences in NUE among different genotypes, and the high NUE genotypes could be selected from the initial plant material. Therefore, one important approach to improve the NUE of crop plants is to develop an understanding of the plant response to N- limitation by comparing two extreme genotypes and using various methods including transcription profiling, mutant analysis, and characterization of plants that grow well under N-limited conditions [5].

Soybean requires more N than other major crops to sustain seed growth [7]. As a legume, soybean can acquire N for its growth via its N-fixing symbiosis with rhizobacteria, which form nodules on the roots and can fix atmospheric N. In addition, soybean can draw mineral nitrogen from the soil. These processes may not supply enough N for soybeans to maximize yield, especially in high-yield environments [8]. With the human population explosion, the energy crisis and environmental pollution, improving the efficiency of N nutrition of plants has become a research hotspot. Therefore, improving the NUE of soybean is a very urgent issue. Genetic engineering is one strategy to enhance the NUE of soybean.

It's necessary to increase the knowledge of soybean gene expression and regulation under N-limited conditions to understand the responses of this crop to different N regimes. Such information is vital for improving the NUE of soybean, and would also be useful to clarify the signal transduction pathways and the mechanism that regulate the N-uptake, assimilation and remobilization pathways.

Next-generation sequencing techniques are opening fascinating opportunities for life sciences, and have dramatically improved the efficiency and speed of gene discovery. This technology can rapidly produce huge numbers of short sequencing reads, making it possible to analyze a complex sample containing a large amount of nucleic acids, by simultaneously sequencing contents of the entire sample [9]. Digital gene-expression (DGE): Tag profiling is a revolutionary approach for expression analysis [10]. Driven by Solexa/Illumina technology, DGE creates genome-wide expression profiles by sequencing. The ability to identify, quantify, and annotate expressed genes on the whole genome level without prior sequence knowledge enables an entirely new scale of biological experimentation, opening doors to higher-confidence target discovery, disease classification, and pathway studies. DGE: Tag profiling also offers researchers a global orthogonal hybridization array validation method, with almost unlimited dynamic range, providing a tunable depth of coverage for rare transcript discovery and quantification. For example, DGE analysis was used to study gene expression in the gastric lymph nodes of Scottish blackface lambs subjected to persistent *Teladorsagia circumcincta *infection [11]. To validate gene expression in the developing digits of two individuals of *Hipposideros armiger*, DGE-tag profiling of developing digits in a pooled sample of two *Myotis ricketti *was analyzed [12]. Age-related autocrine diabetogenic effects of transgenic resistin in spontaneously hypertensive rats were investigated by gene expression profile analysis. This technique has also been used in plant research. Early developing cotton fiber was analyzed by deep-sequencing, and differential expressions of genes in a fuzzless/lintless mutant were revealed [13]. DGE signatures were also used to study maize development, and the results from that study provided a basis for the analysis of short-read expression data and resolved specific expression signatures that will help define mechanisms of action of the maize RA3 gene [14]. In addition, Solexa/Illumina technology was used to analyze gene expression during female flower development [15]. Overall, the DGE approach has provided more valuable tools for qualitative and quantitative gene expression analysis than the previous micro array-based assays.

Here, this is the first genome-wide analysis of gene expression in soybean seedlings under low N stress. Using the Solexa sequencing system, the transcriptomes were compared between seedlings of two soybean varieties, one tolerant and one sensitive to low nitrogen conditions. By investigating the expressions of genes related to N utilization, a number of candidate genes that are important in this process were identified.

## Methods

### Screening soybean varieties for tolerance to low-N conditions

To obtain soybean varieties with different NUEs, 145 varieties were screened (Additional file 1). Soybean seeds were germinated and grown hydroponically in one-half-strength modified Hoagland solution containing 2 mMCa(NO_3_)_2_·4H_2_O,2.5 mM KNO_3_, 0.5 mM NH_4_NO_3_, 0.5 mM KH_2_PO_4_, 1 mM MgSO_4_·7H_2_O, 0.05 mM Fe-EDTA, 0.005 mM KI, 0.1 mM H_3_BO_3_, 0.1 mM MnSO_4_·H_2_O, 0.03 mM ZnSO_4_·7H_2_O, 0.0001 mM CuSO_4_·5H_2_O, 0.001 mM Na_2_MO_4_·2H_2_O, 0.0001 mM CoCl_2_·6H_2_O. The containers used to grow seeds in this solution were 45 × 33 × 20 cm black plastic boxes containing a foam board with 80 holds. This study tested two N levels (N1 level: 10% of the normal N concentration; N2, normal N concentration) in these experiments. The concentration of N in the N1 solution was determined based on a preliminary experiment. Under this N level, stress symptoms (yellow leaves and plant dwarf) were observed within 12 days. The culture solution was refreshed every 3 days. This experiment was conducted once. For preliminary evaluation of N deficiency in soybean plants, the ratios of various parameters, such as relative dry weight, stem length, root length were compared between plants grown in N1 and N2 conditions.

Based on the results of the first screening, three low-N-tolerant varieties and two low-N-sensitive varieties were selected and grown in nutrition solution at two N levels. This experiment was repeated three times. Samples were harvested separately after 0 h and 12 d of treatment. The dry plant weight, stem length, root length and nitrogen content were determined, and these were used as the criteria for screening for genotypes with high NUE. Because different cultivars show genotype-related differences in these biological characteristics, nitrogen use efficiency were estimated using relative indices under several nitrogen levels.

### Plant material and stress treatments

Seeds of the No.116 (low-N-tolerant) and No.84-70(low-N-sensitive) soybean varieties were germinated and grown hydroponically in half-strength modified Hoagland solution. The seedlings were grown for 10 days until the first trifoliate leaves fully developed, and then were grown with 10% of the normal N concentration. The roots and shoots were harvested separately after 0.5, 2, 6 and 12 h, and after 3, 6, 9 and 12d of this treatment. The plant tissues were frozen in liquid nitrogen and kept at -80°C until RNA isolation.

### Solexa/Illumina sequencing

Solexa/Illumina sequencing was carried out by BGI-Shenzhen, China. The main reagents and supplies were the Illumina Gene Expression Sample Prep kit and Illumina Sequencing Chip (flowcell), and the main instruments were the Illumina Cluster Station and the Illumina HiSeq™ 2000 System. The experimental process is summarized as follows: 6 μg total RNA was extracted, and then mRNA was purified with Oligo (dT) magnetic beads. Then, oligo (dT) was used as a primer to synthesize the first and second-strand cDNA. The 5' ends of tags can be generated by two types of endonuclease: *Nla*III or *Dpn*II. Usually, the bead-bound cDNA is subsequently digested with restriction enzyme *Nla*III, which recognizes and removes the CATG sites. The fragments apart from the 3' cDNA fragments connected to Oligo (dT) beads are washed away and the Illumina adaptor 1 is ligated to the sticky 5' end of the digested bead-bound cDNA fragments. The junction of Illumina adaptor 1 and the CATG site is the recognition site of *Mme*I, which has a different recognition and digestion site, i.e., it cuts at 17-bp downstream of the CATG site, producing tags with adaptor 1. After removing 3' fragments with magnetic beads precipitation, Illumina adaptor 2 is ligated to the 3' ends of tags, acquiring tags with different adaptors at both ends to form a tag library. After 15 cycles of linear PCR amplification, 95-bp fragments are purified by 6% TBE PAGE Gel electrophoresis. After denaturation, the single-chain molecules are fixed onto the Illumina Sequencing Chip (Flowcell). Each molecule grows into a single-molecule cluster sequencing template through *in situ *amplification. Then, four types of nucleotides labeled by four colors are added in, and sequencing is performed via the sequencing by synthesis (SBS) method. Each line of the flowcell tunnel will generate millions of raw reads with sequencing lengths of 35 bp.

### Gene expression annotation

All tags were annotated using the database provided by Illumina. Briefly, a preprocessed database of all possible CATG+17-nt tag sequences was created, using the soybean genome and transcriptome. All clean tags were mapped to the reference sequences allowing only a 1-bp mismatch. Clean tags mapped to reference sequences from multiple genes were filtered, and the remaining clean tags were designated as unambiguous clean tags. The number of unambiguous clean tags for each gene was calculated and then normalized to TPM (number of transcripts per million clean tags) [16,17].

### Analysis and screening of differentially expressed genes (DEGs)

Sequencing-received raw image data is transformed by base calling into sequence data, (raw data or raw reads), and is stored in FASTQ format. This type of files stores information about read sequences and quality. Each read is described in four lines in FASTQ files. Raw sequences have 3' adaptor fragments as well as a few low-quality sequences and several types of impurities. Raw sequences are transformed into clean tags after certain data-processing steps. A virtual library was constructed containing all the possible CATG+17 bases length sequences of the reference gene sequences. All clean tags were mapped to the reference sequences and allowing a 1-bp mismatch. Clean tags mapped to reference sequences from multiple genes were filtered. The remaining clean tags were designated as unambiguous clean tags. The number of unambiguous clean tags for each gene was calculated and then normalized to TPM (number of transcripts per million clean tags). A rigorous algorithm [18] was used to identify differentially expressed genes between the two samples. The P-value corresponds to the differential gene expression test. The FDR (False Discovery Rate) is used to determine the threshold of P-value in multiple tests and analyses by manipulating the FDR value. Assume that R differentially expressed genes have been selected, among which S genes truly show differential expression and V genes are false positives. If we decide that the error ratio "Q = V/R" must stay below a cutoff (e.g. 1%), we should preset the FDR to a number no larger than 0.01. FDR ≤ 0.001 and the absolute value of | log2Ratio |≥ 1 were used as thresholds to judge the significance of differences in transcript abundance [19]. More stringent criteria with smaller FDR and greater fold-change value can be used to identify DEGs.

### Real-time quantitative RT-PCR (qRT-PCR) analysis

The expression of candidate genes was determined using qRT-PCR. Tissue samples were removed from the freezer and ground in liquid nitrogen. Total RNA was isolated using Trizol reagent according to the manufacturer's instructions. The quality of the RNA was assessed using an Agilent 2100 Bioanalyzer. The first-strand cDNA fragment was synthesized from total RNA using Superscript II reverse transcriptase (Invitrogen). Gene-specific primers were designed according to gene sequences using Primer 5.0 software. Twenty-four pairs of primers were designed to amplify 24 target genes which were then cloned and sequenced. Using the obtained sequences, gene specific primers were designed for each target gene for qPCR (Additional file 2). Where possible, primers were designed to span intron/exon boundaries to avoid amplification of genomic DNA in qRT-PCR. The quantitative RT-PCR was performed with a iQ™5 and MyiQ™ Real-Time PCR Detection Systems (Bio-Rad) in a final volume of 20 ul containing 2 ul of a 1/10 dilution of cDNA in water, 10 ul 2 × SYBR Green Real-time PCR Master Mix (TOYOBO), and 10 uM of forward and reverse primers. The thermal cycling conditions were as follows: 40 cycles of 95°C for 5 s for denaturation and 55°C for 10 s for annealing and extension. qRT-PCR was performed on three biological replicates. Samples were run in triplicate on the same plate with a negative control that lacked cDNA. Positive controls were set up for each sample in triplicate using soybean the β-actin gene. The soybean β-actin gene was used to normalize gene expressions. PCR efficiency was determined by a series of 2-fold dilutions of cDNAs. The calculated efficiency of all primers was 0.9-1.0. The relative expression levels of genes were calculated using the 2^-ΔCTΔCT ^method, which represents the difference of CT between the control β-actin products and the target gene products.

## Results

### Screening for soybean varieties with high NUE at the seedling stage

To identify soybean varieties with high NUE, a total of 145 varieties were screened at the seedling stage under low-N and normal-N conditions. Relative dry weight, stem length, root length and yellow leaves and fewer tillers were used to evaluate NUE in preliminary screening. From this analysis, we identified three low-N-tolerance varieties (No.108, No.116, and No.165) and two low-N-sensitive varieties (No.166, No.84-70). Further screening were conducted in which were evaluated for other stress tolerance indices; total plant dry weight, ground biomass, total nitrogen accumulation in the shoot and amount of N absorption. There were significant differences among the selected soybean varieties in low-N conditions. As shown in Table 1, among the soybean varieties No.108, No.116, No.165, No.166 and No.84-70, the variety No.116 was the most tolerance to low-N-stress and No.84-70 was the most sensitive.

**Table 1:** Performance of five soybean varieties under low-N and normal-N conditions

Genoty	Dry plant weight			Ground biomass			Total nitrogen accumulation in shoot			Amount of N absorbed		
	
pe	Low N	Normal N	Relative index	Low N	Normal N	Relative index	Low N	Normal N	Relative index	Low N	Normal N	Relative index
108	0.82 b	1.55 a	0.53	0.66 a	1.26 a	0.53	0.94 a	3.23 a	0.29	1.18 a	4.01 a	0.30
165	2.16 a	4.71 b	0.46	1.71 b	2.55 b	0.67	2.87 b	5.72 b	0.50	3.99 b	11.3 b	0.35
116	1.54 a	1.67 a	0.92	1.27 c	1.31 a	0.97	2.38 b	3.10 a	0.77	3.22 b	4.08 a	0.79
166	0.79 b	1.40 a	0.57	0.66 a	1.22 a	0.54	1.22 a	3.02 a	0.40	1.59 a	3.82 a	0.42
84-70	0.35 c	1.52 a	0.23	0.24 d	1.17 a	0.21	0.19 c	4.27 b	0.05	0.41c	5.67 a	0.07

### Sequencing evaluation

**Figure 1:**
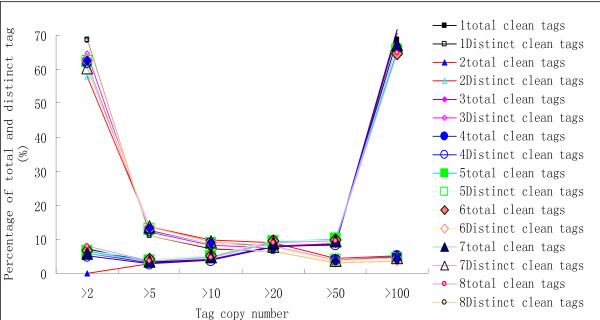
**Distribution of total tag (solid symbols) and distinct tag (open symbols) counts over different tag abundance categories from eight libraries**.

To obtain an overall view of the soybean gene expression profile under low-N conditions, cDNA samples were prepared from No.116 and No.84-70 from 0.5 h to 12 d of a low-N stress treatment. The samples taken at 0.5, 2, 6, and 12 h were selected as the short-term library and those taken at 3, 6, 9, and 12 d as the long-term library. Hence, the following samples were used for sequencing: L1, 116-shoot short-term; L2, 84-70-shoot short-term; L3, 116-shoot long-term; L4, 84-70-shoot long-term; L5, 116-root short-term; L6, 84-70-root short-term; L7, 116-root long-term; and L8, 84-70-root long-term. The Illumina system was used for Tag-sequencing. Expressed genes were identified in No.116 and No.84-70. The number of tags for each library ranged from 5.8 to 6.2 million, and the number of tags producing distinct sequences ranged from 0.3 to 0.5 million (Table 2). The distribution of the various tag abundance categories between total and distinct tag counts showed very consistent results for all libraries (Figure 1). Among the distinct tags, less than 5% had more than 100 copies, 24% of the tags had 5-50 copies, and more than 60% of the tags had 2-5 copies.

**Table 2:** Categorization and abundance of tags

Summary		L1	L2	L3	L4	L5	L6	L7	L8
Raw tag	Total	5986157	6040684	5895889	6168748	5865045	6216559	6198321	6131056
	Distinct tag	466332	351691	351724	375846	591374	504549	519273	525066
Clean tag	Total number	5739999	5846807	5731901	5970775	5476878	5900343	5930716	5862642
	Distinct tag number	224154	162415	191994	181792	204639	206998	233839	257077
All Tag Mapping to Gene	Total number	4801389	4749586	5060517	5106678	4461094	4703005	4633630	4622199
	Total % of clean tag	83.65%	82.86%	86.55%	85.53%	81.45%	79.71%	78.13%	78.84%
	Distinct Tag number	106706	97198	101471	103126	109186	115835	113282	120719
	Distinct Tag % of clean tag	47.60%	50.63%	62.48%	56.73%	53.36%	55.96%	48.44%	46.96%
	Total number	3292723	3227948	3507713	3554706	3256090	3434881	3343733	3402403
	Total % of clean tag	57.36%	56.32%	59.99%	59.54%	59.45%	58.21%	56.38%	58.04%
Unambiguous Tag Mapping to Gene	Distinct Tag number	81596	75187	78233	79048	83632	88491	86619	92556
	Distinct Tag % of clean tag	36.40%	39.16%	48.17%	43.48%	40.87%	42.75%	37.04%	36.00%
	number	37582	36093	35450	35798	37001	36554	36752	38802
	% of ref genes	56.76%	54.51%	53.54%	54.07%	55.88%	55.21%	55.51%	58.60%
All Tag-mapped Genes	number	29503	28271	27960	27977	29174	28799	29217	30581
	% of ref genes	44.56%	42.70%	42.23%	42.25%	44.06%	43.50%	44.13%	46.19%
Unambiguous Tag-mapped Genes	Total number	271	460	131	180	567	396	434	626
	Total % of clean tag	0.00%	0.01%	0.00%	0.00%	0.01%	0.01%	0.01%	0.01%
Mapping to Mitochondrion	Distinct Tag number	39	44	25	32	57	41	38	68
	Distinct Tag % of clean tag	0.02%	0.02%	0.02%	0.02%	0.03%	0.02%	0.02%	0.03%
	Total number	55363	40659	19740	21626	7022	6884	5876	7903
	Total % of clean tag	0.96%	0.71%	0.34%	0.36%	0.13%	0.12%	0.10%	0.13%
Mapping to Chloroplast	Distinct Tag number	580	509	387	478	357	348	284	379
	Distinct Tag % of clean tag	0.26%	0.27%	0.24%	0.26%	0.17%	0.17%	0.12%	0.15%
	Total number	556745	583356	494737	541514	547827	615239	579952	689884
	Total % of clean tag	9.70%	10.18%	8.46%	9.07%	10.00%	10.43%	9.78%	11.77%
Mapping to Genome	Distinct Tag number	76405	59263	38836	48896	47539	44830	45655	77752
	Distinct Tag % of clean tag	34.09%	30.87%	23.91%	26.90%	23.23%	21.66%	19.52%	30.24%
	Total number	326231	357840	271682	300777	460368	574819	710824	542030
	Total % of clean tag	5.68%	6.24%	4.65%	5.04%	8.41%	9.74%	11.99%	9.25%
Unknown Tag	Distinct Tag number	40424	34980	21696	29260	47500	45944	74580	58159
	Distinct Tag % of clean tag	18.03%	18.22%	13.36%	16.10%	23.21%	22.20%	31.89%	22.62%

Clean tags are those remaining after low quality tags have been removed from the raw data. Distinct tags are different kinds of tags. Unambiguous tags are the clean tags remaining after removal of tags mapped to reference sequences from multiple genes.

After filtering dirty tags from raw data, a total of 5,739,999, 5,846,807, 5,731,901, 5,970,775, 5,476,878, 5,900,343, 5,930,716 and 5,862,642 clean tags that corresponded to 224,154, 162,415, 191,994, 181,792, 204,639, 206,998, 233,839 and 257,077 distinct tags for L1, L2, L3, L4, L5, L6, L7 and L8 libraries were obtained, respectively. Eight databases represented expressed sequences (or the transcriptome) for each library. Tags can be mapped to known transcripts to reveal the molecular events behind DGE profiles. In our study, the tag sequences of the eight DGE libraries were mapped to the Soybean (*Glycine max*) genome project, and they matched to more than 80% of all sequence entries in the databases. The tags mapping to the database generated 29,503, 28,271, 27,960, 27,977, 29,174, 28,799, 29,217 and 30,581 tag-mapped transcripts for L1, L2, L3, L4, L5, L6, L7, and L8 libraries, respectively.

### Gene ontology functional enrichment analysis of DEGs

Gene Ontology (GO) is an international standardized gene functional classification system that describes properties of genes and their products in any organism. GO has three ontologies: molecular function, cellular component and biological process. The basic unit of GO is the GO-term. Every GO-term belongs to a type of ontology. In gene expression profiling analysis, GO enrichment analysis of functional significance applies a hypergeometric test to map all DEGs to terms in the GO database, looking for significantly enriched GO terms in DEGs comparing to the genome background. The formula used is as follows:

$$P=1-\overset{m=1}{\underset{i=0}{\sum }}\frac{\left(\begin{array}{c} M\\ i \end{array}\right)\left(\begin{array}{c} N-M\\ n-i \end{array}\right)}{\left(\begin{array}{c} N\\ n \end{array}\right)},$$

where N is the number of all genes with GO annotation; n is the number of DEGs in N; M is the number of all genes that are annotated to the certain GO terms; and m is the number of DEGs in M. The p value is corrected by Bonferroni, and we chose a corrected-p value ≤ 0.05 as the threshold value. The GO term (P ≤ 0.05) is defined as significantly differentially expressed genes enriched GO term. This analysis allowed us to determine the major biological functions of differentially expressed genes.

**Figure 2:**
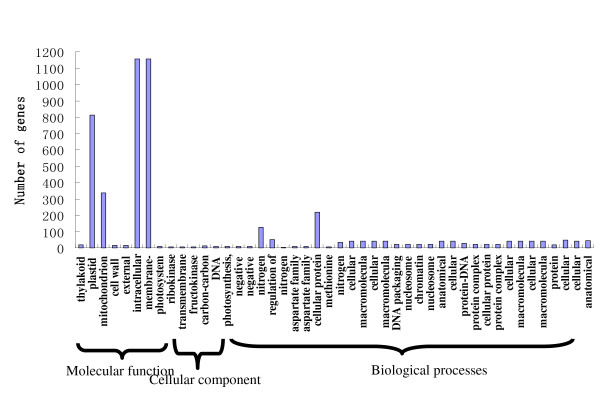
**Histogram showing Gene Ontology functional enrichment analysis of DEGs**.

4,786 differentially expressed genes that could be categorized into 45 functional groups were found (Figure 2). The genes involved in cellular protein complex assembly [GO:0043623] were the most significantly enriched in comparison to the other 44 functional groups. Some DEGs encoded tubulin. Ten DEGs were transcripts encoding products involved in aspartate family amino acid metabolism [GO: 0009066], which included the chemical reactions and pathways involving amino acids of the aspartate family(asparagine, aspartate, lysine, methionine and threonine). Among the significantly enriched transcripts were 51 DEGs associated with regulation of nitrogen compound metabolism [GO: 0051171], which modulates the frequency, rate, or extent of the chemical reactions and pathways involving nitrogen or nitrogenous compounds. In addition, 17 DEGs associated with the cell wall and 21 DEGs associated with protein complex biogenesis were also enriched.

### Pathway enrichment analysis for DEGs

Often, different genes cooperate to achieve their biological functions. Pathway-based analysis helps to further understand the biological functions of those genes. For the pathway-based analysis, KEGG was used, the major public pathway-related database [20]. Pathway enrichment analysis identifies significantly enriched metabolic pathways or signal transduction pathways in DEGs in comparison to the whole genome background. The formula used for this calculation is the same as that used in the GO analysis. Here, N is the number of genes with a KEGG annotation, *n *is the number of DEGs in N, M is the number of genes annotated to specific pathways, and m is the number of DEGs in M. The pathways with a Q value of ≤ 0.05 are defined as those with significantly differentially expressed (enriched) genes. By pathway enrichment analysis we can determine which metabolic and signal transduction pathways the differentially expressed genes are associated with.

**Figure 3:**
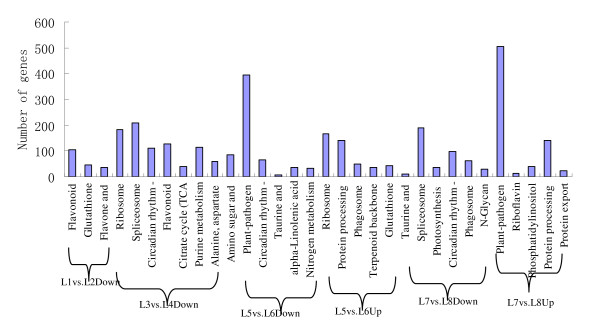
**Histogram illustrating pathway enrichment analyses**.

3,231 differentially expressed genes associated with 22 metabolic and signal transduction pathways were found (Figure 3). The pathways with the most unique sequences were 'metabolic pathways' (1,237 members); 'genetic information processing pathways' (668 members); 'organismal systems pathways' (1,173 members); 'cellular processes pathways' (113 members); and 'environmental information processing pathways' (40 members) (Additional file 3). We believe that these pathways are significant in plants under low-N stress conditions, especially 'metabolism pathways' and 'environmental information processing pathways'. 'Metabolism pathways' are large complexes comprising several metabolism patterns, such as 'amino acid metabolism' [21,22], 'carbohydrate metabolism' [23], 'nitrogen metabolism' [24] and 'biosynthesis of other secondary metabolites' [25]. In this study, some up-regulated and down-regulated genes involved in most stages of nitrogen metabolism were found. Differentially expressed genes were present in four libraries (L1 vs. L2, L3 vs. L4, L5 vs. L6, and L7 vs. L8), namely: 86 down-regulated and 85 up-regulated genes (Additional file 4). Genomic manipulation of these genes might be important for improving NUE in legumes.

### Differential gene expression between the two soybean varieties

**Figure 4:**
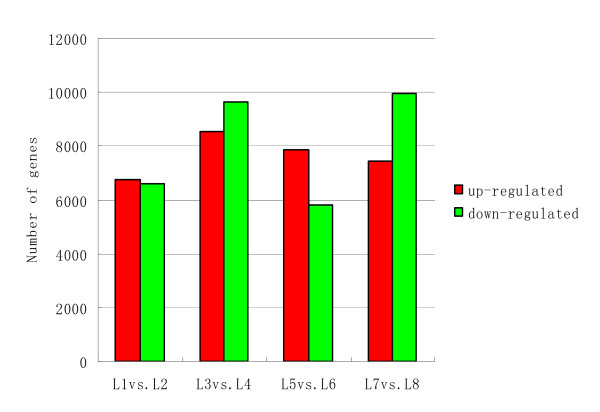
**Changes in gene expression in different samples at various developmental stages**. Numbers of up-regulated and down-regulated genes were summarized.

**Figure 5:**
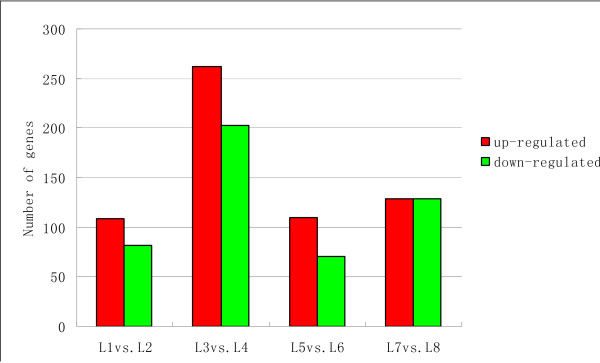
**Number of up-regulated and down-regulated genes with significant differential expressions among different samples**.

Based on "The significance of digital gene expression profiles" [18], a rigorous algorithm was developed to identify genes that were differentially expressed between the two samples. The expression abundance of tag-mapped genes in the data sets was analyzed by counting the number of transcripts per million (TPM) clean tags. First, the read density measurement was normalized as described in detail by Benjamini and Yekutieli [19]. FDR ≤ 0.001 and the absolute value of |log2Ratio|≥ 1 was as thresholds to judge the significance of differences in transcript abundance. Analysis of the eight libraries revealed 26,250, 25,258, 25,181, 25,052, 26,324, 26,299, 26,339, and 27,233 tag-mapped transcripts for L1, L2, L3, L4, L5, L6, L7 and L8, respectively (Additional file 5). Variations in transcript abundance between low-N-tolerance and low-N-sensitive soybean genotype were compared. The results showed that 13,362, 18,165, 13,668, and 17,412 genes showed differential expression levels in L1 vs. L2, L3 vs. L4, L5 vs. L6, and L7 vs. L8, respectively (Figure 4). After filtering against the thresholds of FDR ≤ 0.001 and |log2 Ratio|≧ 1, 191, 465, 180 and 258 genes were detected with significant differential expression levels; These included both up-regulated and down-regulated genes (Figure 5).

**Figure 6:**
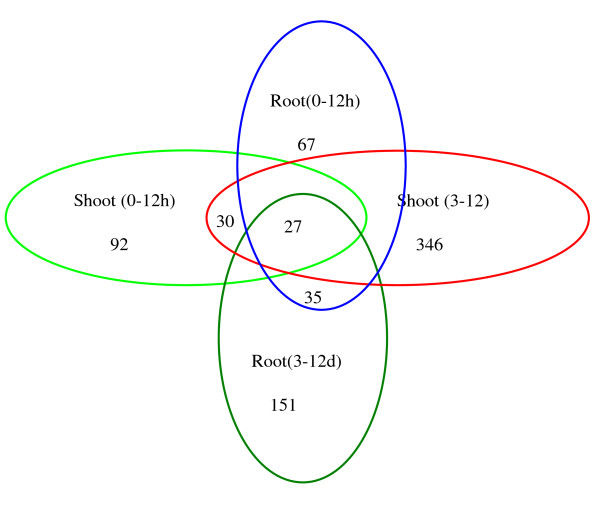
**Analysis of tag-mapped transcripts in eight libraries**.

In addition, in this data set, there were fewer genes that showed differential expression on a short time-scale (0-12 h) than on a long time-scale (3-12 d), suggesting that many genes was up-regulated in growing plants. The DGE results revealed that 92 genes (46 down-regulated and 46 up-regulated) were only found in the shoot in the short-term response, 346 genes (146 down-regulated and 200 up-regulated) were only found in the shoot of long time stage, 67 genes (21 down-regulated and 46 up-regulated) were only found in the root in the short-term response, 151 genes (81 down-regulated and 70 up-regulated) were only found in the root in the long-term response. 27 genes (7 down-regulated and 20 up-regulated) which were differentially expressed in all four stages were also found (Figure 6).

### Functional annotation of differentially expressed genes

After identifying differentially expressed genes, their annotations were established using GO functional enrichment analysis. In addition, all the genes were mapped to terms in the KEGG database, and compared with the complete reference gene background to identify genes involved in pathways that were significantly enriched. Among all the genes with KEGG pathway annotations, 6,473 differentially expressed genes were identified between L1 and L2; 9,014 between L3 and L4; 6,758 between L5 and L6, and 8,628 between L7 and L8. In the four libraries, the main significantly enriched pathways were the plant circadian rhythm pathway, the flavone/flavonol biosynthetic pathway, the glutathione metabolism pathway, the citrate cycle (TCA cycle), the alanine, aspartate and glutamate metabolism pathway, the nitrogen metabolism pathway, the phosphatidylinositol signaling system, and protein export and ribosome pathways. We noted that the 'nitrogen metabolism' pathway was directly involved in nitrogen availability [26]. Large amounts of energy are required to drive the nitrate assimilation, ammonium assimilation and amino acid biosynthesis pathways. The 'carbohydrate metabolism' pathway could provide most of the energy for these pathways [27].

The 10 most differentially expressed genes in each of L1 vs. L2, L3 vs. L4, L5 vs. L6, and L7 vs. L8 libraries are shown in Table 3. The relative abundance is expressed as a TPM ratio of target group: control group. As shown in Table 3, these genes included transcription factors, protein kinases, dehydrogenases, etc.

**Table 3:** Most differentially expressed annotated genes in L1 vs. L2, L3 vs. L4, L5 vs. L6, and L7 vs. L8 libraries based on expressed tag frequency

Gene ID	Relative abundance(TPM ratio)	Functional annotation
L1 vs. L2 up		
Glyma13g17820	1009.41	polyubiquitin
Glyma14g09420	428.92	Papain family cysteine protease
Glyma18g50500	362.37	Unknown
Glyma18g03310	101.05	Hs1pro-1-like receptor
Glyma18g46060	74.04	Rpp4 candidate
L1 vs. L2 down		
Glyma18g51330	25.83	NSP-interacting kinase
Glyma13g36750	17.27	Endocytosis
Glyma08g43410	13.51	BRG-1 ASSOCIATED FACTOR 60
Glyma15g02250	11.63	MYB transcription factor MYB52
Glyma04g37460	11.29	DEK PROTEIN
L3 vs. L4 up		
Glyma02g45690	61.76	similar to PDF
Glyma09g02210	57.05	Serine/threonine protein kinase
Glyma10g40580	33.32	Gibberellin regulated protein
Glyma05g04490	25.12	Protease inhibitor/seed storage/LTP family
Glyma14g05300	24.6	Glycosyl hydrolases family 17
L3 vs. L4 down		
Glyma09g04150	223.25	unknown
Glyma19g38570	94.8	Late embryogenesis abundant protein
Glyma11g37360	35.34	Glyceraldehyde 3-phosphate dehydrogenase
Glyma08g28800	28.14	Ribosomal protein L7Ae/L30e/S12e/Gadd45 family
Glyma06g14330	27.8	Ribosomal protein L6
L5 vs. L6 up		
Glyma03g08020	240.28	ABC transporter related protein
Glyma19g43830	52.95	cyclophilin
Glyma16g26950	41.45	protein-l-isoaspartate methyltransferase
Glyma01g30060	32.87	CBR-TTR-47 protein
Glyma14g02700	26.84	OTU-like cysteine protease family protein
L5 vs. L6 down		
Glyma19g38570	193.57	harpin inducing protein
Glyma18g47100	62.39	conserved hypothetical protein
Glyma04g40170	39.29	methyl esterase 17
Glyma18g09290	36.76	disease resistance protein
Glyma18g45250	34.4	vestitone reductase
L7 vs. L8 up		
Glyma16g24920	106.6	functional candidate resistance protein KR1
Glyma12g04380	62.54	transcription factor homolog BTF3-like protein
Glyma19g36060	40.17	glutathione S-transferase omega
Glyma04g16010	17.8	hypothetical protein
Glyma14g07190	17.63	dehydration-responsive family protein
L7 vs. L8 down		
Glyma18g45250	102.34	NAD dependent epimerase/dehydratase family
Glyma06g14330	79.32	Ribosomal protein L6
Glyma03g03830	61.24	UDP-glucuronosyl and UDP-glucosyl transferase
Glyma01g05800	42.47	membrane associated ring finger 1,8
Glyma05g35490	34.97	predicted protein

### Genes encoding transcription factors

Transcription factors are essential for the regulation of gene expression. Changes in gene transcription are associated with changes in expression of transcription factors. Our DGE results showed that forty-eight genes encoding transcription factors were induced by 1.85 to 62.54-fold, including thirty-one up-regulated and seventeen down-regulated genes. Among the forty-eight genes, six were bHLH family proteins, two were bZIP transcription factors, five were MYB transcription factors, one was a putative TATA element modulatory factor, one was a GT-2 transcription factor, one was a HMG box factor SOX-1, one was a EIL1 transcription factor, one was an auxin response factor, one was a BTF3-like protein transcription factor, and the others were all zinc-finger family proteins.

### Kinases

Kinases play important roles in the development of eukaryotic cells, such as cell cycle control and cell-type determination and differentiation [28]. They regulate metabolic processes in various organs and tissues, and facilitate and control growth, differentiation, reproductive activities, learning and memory. Kinases help the organism to cope with changing conditions and stresses in the environment. Because some of their targets are transcription factors, they also play a role in regulating transcription [29]. Forty-two kinase genes were identified as significantly differentially expressed transcripts, including twenty-four up-regulated and eighteen down-regulated genes. Among these twenty-four genes, four were Tyrosine kinases, nineteen were serine-threonine protein kinases, three were leucine-rich repeat transmembrane protein kinases, two were wall-associated kinases, two were stress-induced receptor-like kinases, and fifteen were other types of kinases.

### Genes involved in carbon and energy metabolism

Many genes involved in carbon and energy metabolism were differentially expressed under low-N conditions. Altered expressions of numerous genes involved in glycolysis, the citrate cycle, oxidative phosphorylation, nitrogen metabolism and photosynthesis were observed. For example, four genes involved in phosphorylation showed increased transcript abundance. These genes encoded casein kinase II subunit alpha (Glyma17g17790), Cdc2-related protein kinase (Glyma04g37630), triose-phosphate transporter family protein (Glyma14g23570) and glucose-6-phosphate 1-dehydrogenase (Glyma19g41450). The TPMs for those transcripts were up-regulated by 3.14 to 5.66-fold. Eight genes involved in photosynthesis were differentially expressed including three genes encoding pfkB family carbohydrate kinases. Their expressions were increased by 2.44-fold(Glyma01g07780), 2.44-fold(Glyma10g32050), and 3.9-fold(Glyma14g37260). Four genes encoded chloroplast-related proteins, including three up-regulated Chlorophyll a/b binding protein genes (Glyma04g08370, Glyma05g24660, and Glyma09g07310) and one down-regulated chloroplast-targeted copper chaperone gene (Glyma03g37060). In addition, one gene encoding a photosynthetic reaction center protein (Glyma13g15560) was up-regulated. In the glycolysis pathway, genes encoding eight glycosyl hydrolase family members were differentially expressed; two were down-regulated, and six were up-regulated with the greatest increase (6.98-fold) observed for Glyma04g01030.

### Nitrogen assimilation-related genes

Nitrogen assimilation is a fundamental biological process in plants. The assimilation of nitrogen has profound effects on plant productivity, biomass, and crop yield, and nitrogen deficiency can inhibit the formation of structural components. Some genes involved in nitrogen assimilation showed significant differential expressions in this study. For example, our DGE results indicated that seven genes encoding amino acid transporter proteins were differentially expressed: four genes were up-regulated (Glyma06g09270, Glyma10g12290, Glyma11g11310, Glyma14g05890) and three genes were down-regulated (Glyma01g36590, Glyma13g44450, Glyma17g26590). In addition, two genes encoding a glutamate synthase family protein (Glyma02g35560) and an asparagine synthetase (Glyma14g37440) were up-regulated; and one nitrate gene (Glyma10g08730) was down-regulated.

### Other differentially regulated genes

There were other genes that showed high-level differential expression related to low-N conditions. After the analysis of the differentially expressed genes in DEGs, six genes related to oxidoreductase activity were identified; a putative ACC-oxidase, a 3-hydroxyacyl-CoA dehydrogenase, a short-chain dehydrogenase and an omega-3 fatty acid desaturase. Six defense response genes were also identified; a putative defensin-like protein, a candidate disease-resistance protein, a wound-induced protein, an abscisic acid-responsive HVA22 family protein, and a GDSL-motif lipase. In addition, one gene encoding a BURP domain protein and one gene encoding a CBS domain-containing protein were found. Another two genes (Glyma08g48240 encoding a UDP-glycosyltransferase and Glyma10g38990 encoding a phosphoinositide binding protein) were also up-regulated. Expression of Glyma10g40580 encoding a gibberellin-regulated protein was up-regulated 33.32-fold under low-N conditions. Expression of Glyma12g33350 encoding an aminotransferase family protein was up-regulated 8.76-fold. Expression of Glyma14g07190 encoding a dehydration-responsive family protein was up-regulated 17.63-fold. Some genes encoding ABC family proteins were also differentially expressed (Glyma19g13500, Glyma08g07560, Glyma06g14450, and Glyma16g28900).

### Confirmation of tag-mapped genes by qRT-PCR

**Figure 7:**
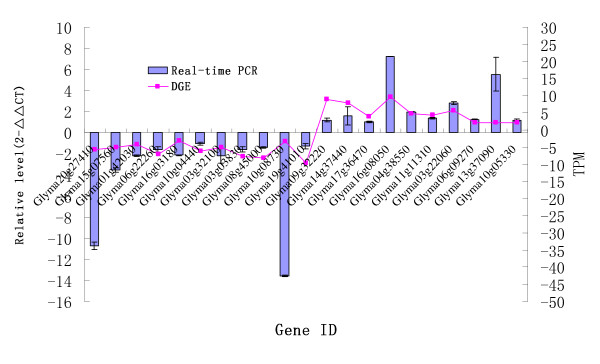
**Real-time PCR validations of tag-mapped genes**.

To confirm the reliability of Solexa/Illumina sequencing technology, twenty-four genes were randomly selected for quantitative RT-PCR assays. The detailing information about individual parameters associated with each step of the RT-qPCR workflow was summarized (additional file 6). The results showed that expressions of twenty-one genes were consistent between the qRT-PCR and the DGE analyses (Figure 7).

## Discussion

This study demonstrated differential transcript abundance and regulation in response to low-N stress between two soybean varieties, one tolerance and one sensitive to low-N conditions. N-stress frequently occurs in agricultural field conditions, and to improve the NUE of plants, it is necessary to formulate strategies to manipulate the genetic architecture of soybean. In this study, numerous genes showed altered expression in plants under low-N stress. These different expressions were analyzed by DGE profiling, which is a fully quantitative approach for gene expression analysis [30]. Identification of differentially expressed genes provides a new platform for understanding the relationships between complex N-responses and regulatory mechanism [31]. Using tag-based deep-sequencing, a direct digital readout of cDNAs can be obtained, showing a dynamic range of genes from transcript libraries. In these experiments, approximately 25,000-27,000 tag-mapped genes were identified for each library. Detailed analysis of N-related genes and pathways showed that approximately 15 significantly differentially expressed genes were enriched in various N-related metabolic or signaling pathways. In addition, several other biological processes that have not previously been linked to N stress, such as flavonoid biosynthesis, natural killer cell mediated cytotoxicity, flavone/flavonol biosynthesis, the phosphatidylinositol signaling system, and N-Glycan biosynthesis, were dramatically altered during N-stress response. These might be novel genes that are relevant to NUE in soybean.

### Nitrogen metabolism genes

Through annotation of the transcriptome and screening for differentially expressed genes, several putatively N-related genes were discovered. These included both up-regulated and down-regulated genes. Nitrogen is utilized by plants in several steps, including uptake, assimilation, translocation, recycling, and remobilization [26]. These events are highly dynamic and complex, and numerous genes are potentially involved.

In plants, N uptake is based on absorption kinetics of transporters across the root cell membranes, mass flow, and diffusion to the surface of single or composite roots. Among the candidate genes identified in this study, some may play roles in the uptake process, such as Glyma13g17730, Glyma17g10440, Glyma05g01450, and Glyma02g43740, all of which are nitrate transporters that are presumably responsible for nitrate absorption from soil [32]. Some genes were related to the cell membrane, where they may play roles in nutrient absorption and/or the N-uptake process. These genes included a wall-associated kinase (Glyma19g21700) and a membrane-associating domain (Glyma16g08050).

Another fundamental biological process that occurs in plants is N assimilation. The major enzymes in N assimilation are glutamine synthetase (GS), glutamate synthase (GOGAT), glutamate dehydrogenase (GDH), aspartate aminotransferase (AspAT), and asparagine synthetase (AS). Each of these enzymes exists in multiple isoenzymic forms encoded by distinct gene families [33]. Several candidate genes that may take part in N-assimilation were found, such as glutamate dehydrogenase (Glyma02g07940), which might play a unique role in assimilating ammonia or catabolizing glutamate during these processes; an NADH glutamate synthase precursor (Glyma14g32500), which was hypothesized to be linked to the process by which NADH-GOGAT catalyzes the rate-limiting step of ammonia assimilation in root nodules; and asparagine synthetase (AS;Glyma14g37440), which is regulated by the carbon/nitrogen status of the plant. The levels of asparagine and AS activities are also controlled by environmental and metabolic signals [34]. In this study, a gene (Glyma12g33350) encoding a predicted aspartate aminotransferase that was up-regulated under low-N conditions was found. In plants, aspartate aminotransferase (AAT, EC2.6.1.1) plays a key role in primary N assimilation, the transfer of reducing equivalents and the interchanges of carbon and nitrogen pools among subcellular compartments [35].

### Regulation of transcription factors and protein kinases

A single transcription factor can regulate expression of multiple genes in a metabolic pathway, and transcription factors are important for regulating many plant responses. Therefore, one approach to genetically improve crops is to modify metabolism pathways. Transcription factors might therefore be potent tools to engineer enhanced stress tolerance in plants [36,37]. Nitrate is the main source of nitrogen for plants, and it serves as the primary signal for several developmental processes including carbon/N metabolism and other metabolic pathways. It is likely that the expressions of numerous genes are regulated in these processes. Some transcription factors and kinases are related to these processes [7]. For example, expressing a Dof1 transcription factor in Arabidopsis improved growth and increased N assimilation under low-N conditions by regulating genes encoding enzymes for production of the carbon skeleton [38]. Therefore, enhanced expression of the key transcription factor(s) could improve the stress tolerance of soybean.

The GATA factors constitute a subgroup of DNA-binding proteins whose members recognize HGATAR core sequences within promoters and enhancers [39]. Many GATA factors can activate or inactivate genes in response to environmental deficiencies and/or to extract chemical elements (i.e., iron, nitrogen, etc.) from the surrounding environment. Some GATA factors regulate N metabolism and are required to activate expression of N catabolic enzymes during periods of N- deficiency in fungi [40]. However, little is known about the functions of GATA factors in plants. In this study, a gene encoding a hypothetical GATA factor protein (Glyma12g08130) showed differential expression under low-N condition. We assume that this gene is involved in N- assimilation in soybean. The function of the gene will be studied by RNA-interference or by overexpression in transgenic plants in the near future.

Several lines of biochemical and genetic research indicate that reversible protein phosphorylation is involved in the regulation of plant stress responses to various environmental stimuli. Some protein kinases might be involved in the regulation of cell differentiation and N-metabolism in nitrogen-fixing filamentous cyanobacteria [41,42]. Wall-associated kinases are also involved in various processes in plants, including pathogen resistance, heavy-metal tolerance and organ development [43]. Unfortunately, little is known about their function in tolerance to nutrient deficiency. Our DGE results indicated that two genes encoding wall associated kinases, Glyma19g21700 and Glyma19g21690, were up-regulated under N-limited conditions. In addition, a gene encoding receptor-like kinase (Glyma13g09810) was differentially expressed between the two varieties under N-limited conditions. Recent studies revealed that higher plants also have genes encoding putative receptor kinases (receptor-like Kinases; RLKs). For instance, the completely sequenced Arabidopsis genome contains more than 500 genes encoding RLKs, suggesting that higher plants, like animals, use receptor kinase signaling widely to modulate expressions of genes in response to diverse stimuli. Some research indicated that receptor-like kinases (RLK) play important roles in plant growth and development as well as in hormone and stress responses [44]. Therefore, we hypothesize that the Glyma13g09810 gene might be important for adaptation to low-N conditions in soybean.

### Other differentially regulated genes

In addition to the genes described above, several other transcript profiles were altered under low N conditions. For example, a gene encoding BURP domain protein (Glyma04g35360) was differentially expressed. Some reports suggest that genes from the BURP family may be crucial for responses and adaptations to stresses. All the members of this family were shown to be induced by at least one type of stress treatment, for example, drought, salt, cold, abscisic acid and nutrition, etc. [45]. Therefore, the soybean BURP gene may be N responsive to N-stress. One gene encoding CBS domain-containing protein which was differentially expressed in two soybean varieties was also found. Previous research revealed that CBS domain-containing proteins play important roles in stress response/tolerance and development in plants [46]. To determine whether this protein has the potential to improve tolerance of transgenic plants to low N-stress, its role in development and N stresses should be further investigated. In addition, some published results suggest that a phosphatase is involved in modulating phosphoinositide signals during the stress response [47]. This results showed that one gene (Glyma10g38990) putatively encoding a phosphoinositide binding protein was up-regulated. We suggest that this gene may function as a component of a stress response pathway that protects the plant against the effects of N-deficiency.

The DGE results indicated that three genes predicted to be members of the ABC1 family, were differentially expressed between N1 and N2 conditions. Several plant ABC1 genes participate in the abiotic stress response [48]. Plants have evolved diverse adaptive physiological and biochemical mechanisms to resist various stresses, and thus, expressions of many related genes are altered.

In the DGE analysis of differentially expressed genes under low-N conditions, fifty-three up-regulated and forty-seven down-regulated genes that were not annotated were found. We hypothesize that these genes are putatively N-related transcripts. However, they may be unique to soybean, and therefore, absent from other species. Further research focusing on these genes will be carried out based on the DEGs information and bioinformatics.

## Conclusions

This study has demonstrated the usefulness of the digital gene expression (DGE) approach to identify differentially expression genes between two soybean genotypes in N-limiting conditions. A large data set of tag-mapped transcripts were obtained, which provide a strong basis for future research on the N-nutrition of other crops. In addition, a new list of candidate targets for functional studies on genes involved in N utilization has been generated. Further work should concentrate on characterizing these genes. This could lead to a better understanding of the genetic basis of the phenotypic differences between the two soybean genotypes in N-limiting conditions. This is essential for improving the NUE of soybean.

## Authors' contributions

HQN designed the study, managed plants, isolated samples, analyzed data, performed qRT-PCR, and wrote and revised the manuscript; XAZ designed the study and critically reviewed the manuscript; AHS coordinated and helped to draft the manuscript and critically reviewed manuscript; CW helped to do some experiment; ZR, provided soybean seeds for research; SLC prepared reagents. All the authors read and approved the final manuscript.

## Supplementary Material

Additional file 1 **List of the soybean varieties for screening**. 145 soybean varieties from different areas of China for screening high NUE variety and low NUE variety were listed. Click here for file

Additional file 2 **Primer details for genes selected for RT-PCR analysis from results of DGE**. This is the primer list of twenty-four genes which were randomly selected for quantitative RT-PCR assays to confirm the reliability of Solexa/Illumina sequencing technology. Click here for file

Additional file 3 **Pathway enrichment analyses of differentially expressed genes**. Pathway enrichment analyses of differentially expressed genes were summarized from four libraries (L1 vs. L2, L3 vs. L4, L5 vs. L6, and L7 vs. L8). Click here for file

Additional file 4 **Differentially expressed genes involved in nitrogen metabolism**. The differentially expressed genes involved in nitrogen metabolism were summarized from four libraries (L1 vs. L2, L3 vs. L4, L5 vs. L6, and L7 vs. L8): 86 were down-regulated and 85 were up-regulated. Click here for file

Additional file 5 **Gene expression analyses of eight libraries**. The details of gene expression analyses of L1, L2, L3, L4, L5, L6, L7, and L8 libraries: Gene, Synonyms, GeneExpression, TPM, GO Component, GO Function, GO Process, blast nr, transcriptID, Mapped-Tag, Nth-Tag-from-3'-end-of-Gene, Tag-Copy-Number and TPM(tag). Click here for file

Additional file 6 **The RT-qPCR workflow**. Detailing information about individual parameters associated with each step of the RT-qPCR workflow. Click here for file
