# Supplementary material for: Identification of genes associated with nitrogen-use efficiency by genome-wide transcriptional analysis of two soybean genotypes
Source: BMC Genomics. 2011 Oct 26;12:525. doi: 10.1186/1471-2164-12-525 (PMC3210170; doi:10.1186/1471-2164-12-525)
Supplement: Additional file 1 — List of the soybean varieties for screening. 145 soybean varieties from different areas of China for screening high NUE variety and low NUE variety were listed. [file 1471-2164-12-525-S1.DOC]

| ZhongDou35  XiangchuDou10  EDou4  You96-5  Jitouxuan Blackbeans  TaiXingBlackbeans LiangJiangPosoybean  TaiXingAiJiaoHong  SuiNingFengTaiJiangSe  YouChun 05-8  You03-68  PiXianruantiaozhi  L73-1018  ZaDou-6  L69-4667  You01-65  XuDou 9  Jiang soybean  Peixianxiaoyoudou  Pixianlayanghuang  ZhongDou8  L71-1388  L73-1034  Green seed coat soybean  Camp  LongDou7  YouWuDou  QiongLaiXiJiangBlack Bean  ShanZiBai  05-4 | XiangChunDou18  XiangChunDou22  XiangChunDou 23  YouChun 01-45  EDou 7  HengFengWuDou  FengChengZaoWuDou  LongChuanHuangNiuMao  DuChangWuDou  MaDaiHeiDou-3  L74U-6710  L70-4313  L68-1774  L76-2100  GongDou13  L81-4651  ZhongPin03-5359  ZhongPin 03-5361  T260H  74-424  L64-314  L67-1749  L70-4186  BaiZhiDou  L70-4558  QuanDou 253  GongDou7  TongShanTianEDan  WuJiangWuYueNiuMaoHuang  HeiHe48 | HongHuLiuYueBao  ZheChun2  NiDou  HuaSeDou  XiaMenTengZaiDou  MaDaiQingDou-2  84-70  DaSoybean-1  PengShanHuangKeZi-3  LiuYueHuang  ZhongPin03-5367-2  Perrin  L68-1864  JianWeiQuanShuiDou  L70-4629  L63-2999  L76-1113  L63-1677  L76-1149  L70-4049  GuiChun1 L77-2654  YiZhengDaLiHuangDou ZhongPin 03-5364  L67-3479  XiaoHeiDou  GuiChun2  XuDou8  XiHuangDou-9 | SuiningPingDingHuang  DaHuangDou-2  PiXianXiaoHuangDou  EJiZaoDou-2  TongAnZiHongDou  ZiZhongLiuYueZao  WuYueHuang  ZaoJiaoDou  AiJiaoZao  ZheChun3  L69-4755  L62-1027  L70-4422  L73-1087  L74-838  L75-6631  ShiFangLuoSiDou  ChangShouShiYueHuang  HuaXia3  ShaXianWuDou  L63-2346  You02-33  ZhongPin03-5355  TongShanQingDaDou  L69-4662  ZhongPin03-5381  DaHungDou  DaLiHuang  L63-1792 | 8307-8-1  PuDou51  ZhongDou32  DongNong50  Lamar（Ⅵ）  XiaoKeHuangDou  QiYueHuang-1  GuiXiaDou2  Suwon165  ZhongPin03-5366  YueChun03-3  ZhongPin03-5412  ZhongPin03-5413  ZhongPin03-5358  Shinpaldal kong2  ShangRaoBaYueBai  DaiMiDou  L67-3124  LaoShuPi  ChaHuangDaiDou1  L72-2004  Suwon164  DaBaiMaoDou  HuiChaXiaoHuangDou  PoHuang（116）  Sharkey  HuaChun2  JianGeHuaLinJiWo  You06-71 |
| --- | --- | --- | --- | --- |
