# Supplementary material for: Identification of genes associated with nitrogen-use efficiency by genome-wide transcriptional analysis of two soybean genotypes
Source: BMC Genomics. 2011 Oct 26;12:525. doi: 10.1186/1471-2164-12-525 (PMC3210170; doi:10.1186/1471-2164-12-525)
Supplement: Additional file 6 — The RT-qPCR workflow. Detailing information about individual parameters associated with each step of the RT-qPCR workflow. [file 1471-2164-12-525-S6.DOC]

The RT-qPCR workflow

| Sample/Template | details |
| --- | --- |
| Source | Soybean [tissue](app:ds:tissue) |
| Method of preservation | Liquid N_2_ |
| Handling | frozen |
| Extraction method | TriZol |
| RNA: DNA-free | Intron-spanning primers |
| Concentration | Agilent 2100 |
| RNA: integrity | Agilent 2100 |
| Inhibition-free |  |

Assay optimisation/validation

| Accession number | Glyma20g27410,Glyma15g07560,Glyma01g42030,Glyma06g22260,Glyma16g03180  Glyma10g04440,Glyma03g32100,Glyma03g03830,Glyma08g45000,Glyma10g08730  Glyma19g41010,Glyma09g32220,Glyma14g37440,Glyma17g36470,Glyma16g08050  Glyma04g38550,Glyma11g11310,Glyma03g22060,Glyma06g09270,Glyma13g37090  Glyma10g05330 |
| --- | --- |
| Primer sequence | supplementary material 4 |
| In silico | Blast |
| empirical | DNA sequencing |
| Priming conditions | oligo-dT |
| PCR efficiency | 90%-100% |
| Linear dynamic range | Y = -3.138*LOG(X) + 45.73,etc. |

RT-PCR

| Protocols | A 20-µl reaction volume can be used for 3 µg of total RNA, 1. Add the following components to a nuclease-free microcentrifuge tube: Oligo(dT)12-18 (500 µg/ml):1ul, 3 µg total RNA :x µl, dNTP Mix (10 mM each): 1ul, Sterile, distilled water to 12 µl. 2. Heat mixture to 65°C for 5 min and quick chill on ice. Collect the contents of the tube by brief centrifugation and add: 5X First-Strand Buffer:4µl, 0.1 M DTT:2µl, RNaseOUT™ (40 units/µl):1ul. 3. Mix contents of the tube gently, incubate at 42°C for 2 min. 4. Add 1 µl (200 units) of SuperScript™ II RT and mix by pipetting gently up and down. 5. Incubate at 42°C for 50 min.6. Inactivate the reaction by heating at 70°C for 15 min. |
| --- | --- |
| Reagents | Superscript II reverse transcriptase (Invitrogen), Cat. Nos. 18064-022 |
| Duplicate RT |  |
| NTC | N/A |

Data analysis

| Specialist software | Bio-Rad iQ5 |
| --- | --- |
| Statistical justification | biological replicates |
| Transparent, validated normalization | 2^−△CT△CT^ |
